# Supplementary material for: Resting natural killer cells promote the progress of colon cancer liver metastasis by elevating tumor-derived stem cell factor
Source: eLife. 2024 Oct 10;13:RP97201. doi: 10.7554/eLife.97201 (PMC11466454; doi:10.7554/eLife.97201)
Supplement: Supplementary file 1. [file elife-97201-supp1.docx]

Table 1. Characterized genes for Single-cell transcriptomic analysis

| **T cells** | **NK cells** | **Native B cell** | **Monocyte** | **Plasma cell** | **Neutrophil cell** | **Monocyte CD16-** | **Monocyte CD16+** |
| --- | --- | --- | --- | --- | --- | --- | --- |
| IL7R | NKG7 | CD79A | SPP1 | IGKC | CXCL8 | S100A9 | C1QA |
| LTB | CCL5 | MS4A1 | APOE | JCHAIN | G0S2 | VCAN | MARCO |
| SPOCK2 | KLRD1 | HLA-DRA | APOC1 | IGLC2 | C15orf48 | S100A8 | C1QB |
| KLRB1 | XCL2 | VPREB3 | TIMP1 | IGHA1 | FCGR3B | LYZ | SELENOP |
| TRAC | CMC1 | BANK1 | IL1B | IGHA2 | NAMPT | S100A12 | PDK4 |
| SARAF | GZMB | IGHD | CXCL2 | IGLC3 | S100A8 | FCN1 | CST3 |
| LDHB | GZMH | CD83 | IFI30 | IGHG1 | BCL2A1 | MNDA | CD5L |
| RORA | CST7 | CD74 | C1QC | IGHG3 | S100A9 | THBS1 | SLC40A1 |
| NDFIP1 | GZMA | HLA-DQB1 | CXCL3 | IGHG4 | CSF3R | CYP1B1 | VCAM1 |
| CD2 | IFNG | MEF2C | LYZ | MZB1 | SOD2 | CTSS | C1QC |
| LEPROTL1 | GZMK | HLA-DPB1 | CTSD | SSR4 | MNDA | GCA | MS4A7 |
| CD3D | CCL4 | CD37 | CST3 | DERL3 | SMIM25 | CD14 | MS4A6A |
| TPT1 | CTSW | TNFRSF13C | FTL | TNFRSF17 | SLC25A37 | IL1R2 | CD163 |
| RPS3A | GZMM | HLA-DQA1 | C1QB | SEC11C | PTGS2 | CD163 | LIPA |
| CD3E | CD8B | HLA-DPA1 | CCL3 | XBP1 | IFITM2 | PLBD1 | SDC3 |
| RPS20 | PRF1 | HLA-DRB1 | C1QA | HSP90B1 | CMTM2 | SERPINA1 | CTSB |
| GPR183 | CD8A | CD79B | HLA-DRA | PRDX4 | CCL3L1 | CSTA | FCGRT |
| TRBC2 | HCST | LINC00926 | CTSB | HERPUD1 | ALOX5AP | SLC11A1 | FTL |
| RPL22 | PIK3R1 | RALGPS2 | HLA-DRB1 | TXNDC5 | LST1 | FPR1 | HMOX1 |
| RPL34 | CCL4L2 | LY9 | CCL3L1 | FKBP11 | NEAT1 | SERPINB1 | CFD |
