# Supplementary material for: Resting natural killer cells promote the progress of colon cancer liver metastasis by elevating tumor-derived stem cell factor
Source: eLife. 2024 Oct 10;13:RP97201. doi: 10.7554/eLife.97201 (PMC11466454; doi:10.7554/eLife.97201)
Supplement: Supplementary file 2. [file elife-97201-supp2.docx]

Table 2. Characterized genes for spatial transcriptomic analysis

| **Tumor** | **Normal epithelium** | **Hepatocytes** | **Lamina propria** | **Fibroblast** | **Smooth muscle** |
| --- | --- | --- | --- | --- | --- |
| SCD | SELENOP | APOA1 | JCHAIN | COL1A1 | CSRP1 |
| RPL4 | CKB | APOA2 | DERL3 | COL4A1 | AOC3 |
| LRRC75A | TSPAN1 | ALB | MZB1 | COL3A1 | TPM2 |
| FABP1 | MT-CO1 | HP | IGHA1 | SPARC | MYL9 |
| CSTB | ZG16 | FGB | IGHG4 | COL5A1 | MYH11 |
| GGCX | SLC26A2 | RBP4 | TXNDC5 | COL4A2 | SELENOM |
| VEGFA | KRT19 | FGG | IGHA2 | C3 | CNN1 |
| ID1 | FCGBP | AHSG | IGHG1 | MCAM | TAGLN |
| TNFRSF6B | SDCBP2 | CYP3A4 | IGHG3 | IGFBP7 | FLNA |
| RPL34 | HLA-C | APOC3 | IGKC | CD93 | ACTG2 |
| S100A6 | B2M | AMBP | CD27 | ENG | ACTA2 |
| HMGCS1 | HLA-B | ORM1 | C3AR1 | COL5A2 | GREM2 |
| ZFAS1 | PLAC8 | CYP2E1 | TRAC | FSTL1 | DES |
| TGFBI | ADM | HRG | TYMP | CTSK | CALD1 |
| IFITM1 | MXD1 | GC | LTB | SERPINF1 | FLNC |
| CCDC88B | ITLN1 | A1BG | JAK3 | MMP11 | MYLK |
| ATP1B1 | GUCA2A | FGA | TRBC2 | MMP2 | SYNPO2 |
| RPL22L1 | MALL | ORM2 | TBC1D10C | C1R | ANGPTL2 |
| CD55 | SECTM1 | ST6GAL1 | CORO1A | COL1A2 | TNS1 |
| AC092069.1 | MYO15B | MT-ATP8 | CXCR4 | HSPG2 | PDE5A |
