# Supplementary material for: Resting natural killer cells promote the progress of colon cancer liver metastasis by elevating tumor-derived stem cell factor
Source: eLife. 2024 Oct 10;13:RP97201. doi: 10.7554/eLife.97201 (PMC11466454; doi:10.7554/eLife.97201)
Supplement: Supplementary file 3. [file elife-97201-supp3.docx]

Table 3. Characterized genes of NK subsets

| **Cluster 0** | **Cluster 1** | **Cluster 2** | **Cluster 3** | **Cluster 4** | **Cluster 5** | **Cluster 6** | **Cluster 7** |
| --- | --- | --- | --- | --- | --- | --- | --- |
| GZMK | FGFBP2 | EEF1G | FCER1G | MTRNR2L12 | IGHA1 | MT-ND6 | CXCL13 |
| IL7R | GNLY | EGR1 | CCL3 | PLCG2 | IGKC | PPP1R1B | CTLA4 |
| TUBA4A | FCGR3A | RPL17 | AREG | HLA-DRB5 | LDLRAD4 | RNF19A | RBPJ |
| CD8A | PTGDS | RNASEK | TYROBP | PSMB9 | IGHA2 | EZR | DUSP4 |
| CXCR4 | GZMB | ATP6V0C | CEBPD | XCL2 | IGLC2 | HLA-DQA1 | TNFRSF18 |
| CD8B | SPON2 | KLRK1 | XCL1 | HIST1H4C | IL7R | SLC2A3 | GZMB |
| RGCC | PRF1 | LIME1 | KLRF1 | AC007952.4 | ITGA1 | CREM | PHLDA1 |
| RPS20 | MYOM2 | HSPA1B | KLRB1 | RPS26 | SPRY1 | METRNL | LINC01480 |
| RPS2 | NKG7 | JUN | NFKBIA | HCST | JCHAIN | PTPRC | SRGAP3 |
| RPL13A | CLIC3 | EIF4A1 | CLIC3 | CD160 | TMIGD2 | PDE4B | LAYN |
| TRGC2 | EFHD2 | AL627171.2 | GADD45B | MTRNR2L8 | CD55 | LCP1 | FAM3C |
| RPL23A | LAIR2 | NME2 | CMC1 | GIMAP7 | CD52 | NCL | SAMSN1 |
| HLA-DPB1 | GZMH | FOSB | IRF8 | MYL12A | ANKRD28 | YWHAZ | TNFRSF9 |
| ZFP36L2 | PLAC8 | CD69 | IL2RB | ITGB2 | MT-CYB | IGKC | SNX9 |
| HLA-DRB1 | TYROBP | NR4A1 | TXK | Z93241.1 | CAPG | IGHG3 | NR3C1 |
| YBX3 | ADGRG1 | KLRC3 | GSTP1 | TMEM107 | PDE4D | TPM3 | ENTPD1 |
| LYAR | CD247 | MIF | KLRC1 | PSME2 | SMIM3 | REL | CXCR6 |
| RPS16 | CX3CR1 | CRIP1 | CD160 | ATP5F1E | LINC01871 | GRB7 | CREM |
| TRAT1 | KLRF1 | DNAJB1 | GRASP | CORO1A | IGLC3 | YPEL5 | CCL20 |
| CRTAM | HOPX | BCL11B | TLE1 | PLEKHF1 | TNFAIP3 | HIST1H1E | GAPDH |
