# Supplementary material for: Resting natural killer cells promote the progress of colon cancer liver metastasis by elevating tumor-derived stem cell factor
Source: eLife. 2024 Oct 10;13:RP97201. doi: 10.7554/eLife.97201 (PMC11466454; doi:10.7554/eLife.97201)
Supplement: Supplementary file 4. [file elife-97201-supp4.docx]

Table 4. Characterized genes of NK of different status

| **Resting NK cell** | **Activated NK cell** | **Other NK cell** |
| --- | --- | --- |
| MTRNR2L12 | EGR1 | CXCL13 |
| FGFBP2 | IGHA1 | CTLA4 |
| TYROBP | EEF1G | RBPJ |
| FCER1G | IGKC | DUSP4 |
| NKG7 | RNASEK | TNFRSF18 |
| FCGR3A | ATP6V0C | GZMB |
| CLIC3 | RPL17 | PHLDA1 |
| KLRF1 | LDLRAD4 | LINC01480 |
| GZMK | IGHA2 | SRGAP3 |
| PLAC8 | KLRK1 | LAYN |
| SPON2 | FOSB | FAM3C |
| PLCG2 | LIME1 | SAMSN1 |
| RPS26 | ITGA1 | TNFRSF9 |
| CMC1 | MT-ND4L | SNX9 |
| ITGB2 | JUN | NR3C1 |
| CD247 | TNFAIP3 | ENTPD1 |
| CST7 | AL627171.2 | CXCR6 |
| GNLY | EIF4A1 | CREM |
| CCL3 | LINC02446 | CCL20 |
| HSPA6 | MT2A | GAPDH |
