# Supplementary material for: Resting natural killer cells promote the progress of colon cancer liver metastasis by elevating tumor-derived stem cell factor
Source: eLife. 2024 Oct 10;13:RP97201. doi: 10.7554/eLife.97201 (PMC11466454; doi:10.7554/eLife.97201)
Supplement: Supplementary file 5. [file elife-97201-supp5.docx]

Table 5. Fluorescence Minus One control of CD56, CD9, PD-1 and CD49a.

| Group | CD56-APC | CD9-FITC | CD49a-PerCP | PD-1-PE |
| --- | --- | --- | --- | --- |
| CD56-FMO | - | + | + | + |
| CD9-FMO | + | - | + | + |
| CD49a-FMO | + | + | - | + |
| PD-1-FMO | + | + | + | - |
