# Supplementary material for: Resting natural killer cells promote the progress of colon cancer liver metastasis by elevating tumor-derived stem cell factor
Source: eLife. 2024 Oct 10;13:RP97201. doi: 10.7554/eLife.97201 (PMC11466454; doi:10.7554/eLife.97201)
Supplement: Supplementary file 6. [file elife-97201-supp6.docx]

Table 6. Fluorescence Minus One control of KIR2DL4 and GZMK.

| Group | CD56-APC | KIR2DL4-PE | GZMK -FITC |
| --- | --- | --- | --- |
| KIR2DL4-FMO | + | - | + |
| GZMK -FMO | + | + | - |
