# Supplementary material for: Resting natural killer cells promote the progress of colon cancer liver metastasis by elevating tumor-derived stem cell factor
Source: eLife. 2024 Oct 10;13:RP97201. doi: 10.7554/eLife.97201 (PMC11466454; doi:10.7554/eLife.97201)
Supplement: Supplementary file 7. [file elife-97201-supp7.docx]

Table 7. Primers for qPCR.

| Target gene | Sequence |
| --- | --- |
| KITLG Forward primer | CAGAGTCAGTGTCACAAAACCATT |
| KITLG Reverse primer | TTGGCCTTCCTATTACTGCTACTG |
| GAPDH Forward primer | GGGGAGCCAAAAGGGTCATCATCT |
| GAPDH Reverse primer | GACGCCTGCTTCACCACCTTCTTG |
