# Supplementary material for: Resting natural killer cells promote the progress of colon cancer liver metastasis by elevating tumor-derived stem cell factor
Source: eLife. 2024 Oct 10;13:RP97201. doi: 10.7554/eLife.97201 (PMC11466454; doi:10.7554/eLife.97201)
Supplement: Source code 1. [file elife-97201-code1.zip › eLife-VOR-RA-2024-97201/Code.docx]

setwd('/home/medie/Rfile/chenchen/CCLM/2.rda')

options(stringsAsFactors = F)

#加载R包

library(Seurat)

library(dplyr)

library(ggplot2)

library(magrittr)

library(gtools)

library(stringr)

library(Matrix)

library(tidyverse)

library(patchwork)

library(data.table)

library(RColorBrewer)

library(ggpubr)

library(data.table)

library(RColorBrewer)

library(ggpubr)

library(SingleR)

library(celldex)

library(BiocParallel)

library(viridis)

library(pheatmap)

library(viridisLite)

load('CCLM.RData')

CCLM<-Seurat_object

setwd('/home/medie/Rfile/chenchen/CCLM/3.jiangju')

library(Seurat)

library(dplyr)

library(ggplot2)

library(magrittr)

library(gtools)

library(stringr)

library(Matrix)

library(tidyverse)

library(patchwork)

library(data.table)

library(RColorBrewer)

library(ggpubr)

CCLM <- NormalizeData(CCLM, normalization.method = "LogNormalize", scale.factor = 10000)

CCLM <- FindVariableFeatures(CCLM,

selection.method = "vst",

nfeatures = 2000,

mean.cutoff=c(0.0125,3),

dispersion.cutoff =c(1.5,Inf))

top20 <- head(VariableFeatures(CCLM), 20)

plot1 <- VariableFeaturePlot(CCLM)

plot2 <- LabelPoints(plot = plot1, points = top20, repel = TRUE, size=3.0)

feat_20 <- CombinePlots(plots = list(plot1, plot2),legend="bottom")

feat_20

ggsave(filename = 'feat_20.pdf',plot = feat_20,he=10,wi=15)

#ScaleData

scale.genes <- rownames(CCLM)

CCLM <- ScaleData(CCLM, features = scale.genes)

CCLM <- RunPCA(CCLM, features = VariableFeatures(CCLM))

dimplot1 <- DimPlot(CCLM, reduction = "pca")

dimplot1

ggsave(filename = 'dimplot2.pdf',plot = dimplot1,he=10,wi=15)

elbowplot1 <- ElbowPlot(CCLM, ndims=50, reduction="pca")

elbowplot1

sc_pca <- dimplot1+elbowplot1

sc_pca

ggsave(filename = 'sc_pca.pdf',plot = sc_pca,he=10,wi=15)

top20 <- VizDimLoadings(CCLM, dims = 1:2, nfeatures = 20, reduction = "pca")

top20

ggsave(filename = 'top20.pdf',plot = top20,he=10,wi=15)

DimHeatmap(CCLM, dims = 1:20, cells = 500, balanced = TRUE)

CCLM$orig.ident

Dims <- 30

Resolution <- 0.1

CCLM <- FindNeighbors(object = CCLM, dims = 1:Dims)

CCLM <- FindClusters(object = CCLM, resolution = Resolution)

allcolour=c("#FF34B3", "#BC8F8F", "#20B2AA", "#00F5FF", "#FFA500", "#ADFF2F", "#FF6A6A", "#7FFFD4","#AB82FF", "#90EE90", "#00CD00", "#008B8B", "#6495ED", "#FFC1C1", "#CD5C5C", "#8B008B", "#FF3030", "#7CFC00", "#000000", "#708090", "#DC143C","#0000FF","#20B2AA","#FFA500","#9370DB","#98FB98","#F08080","#1E90FF","#7CFC00","#FFFF00","#808000","#FF00FF","#CCCCFF","#000000","#7B68EE","#9400D3","#A0522D","#800080","#D2B48C","#D2691E","#87CEEB","#40E0D0","#5F9EA0","#FF1493","#0000CD","#008B8B","#FFE4B5","#8A2BE2","#228B22","#E9967A","#4682B4","#32CD32","#F0E68C","#FFFFE0","#EE82EE","#FF6347","#6A5ACD","#9932CC","#8B008B","#8B4513","#DEB887")

allcolour=c("#E64B35FF", "#4DBBD5FF", "#00A087FF", "#3C5488FF",

"#FFA500", "#8491B4FF", "#FA8072", "#DC0000FF",

"#7E6148FF", "#B09C85FF","#800000","#91D1C2FF")

length(table(sce@active.ident))

mycolor = allcolour[1:length(table(sce@active.ident))]

### UMAP

CCLM <- RunUMAP(CCLM, dims=1:Dims, reduction="pca")

CCLM <- RunTSNE(CCLM,

dims=1:Dims,

reduction="pca")

sc_umap = DimPlot(CCLM,#cols=allcolour,

reduction="umap",

#reduction="tsne",

label = "F",

pt.size = 0.2,

label.size = 3) +

theme(axis.line = element_line(size=0.1, colour = "black"),

axis.ticks = element_blank()

)

sc_umap

ggsave('sc_umapcluster.pdf',sc_umap,he=7,wi=7)

sc_umap_sample = DimPlot(CCLM,#cols=allcolour,

group.by='tissueunique',

#reduction="umap",

reduction="tsne",

label = "T",

pt.size = 1.0,

label.size = 0) +

theme(axis.line = element_line(size=0.1, colour = "black"),

axis.ticks = element_blank()

) +ggtitle('')

sc_umap_sample

ggsave('sc_umap_sample.pdf',sc_umap_sample,he=7,wi=7)

sc_umap_group = DimPlot(CCLM,group.by='Group1',

#reduction="umap",

reduction="tsne",

label = "T",

pt.size = 0.2,

label.size = 0) +

theme(axis.line = element_line(size=0.1, colour = "black"),

axis.ticks = element_blank()

) +ggtitle('')

sc_umap_group

ggsave('sc_umap_group.pdf',sc_umap_group,he=7,wi=7)

setwd('/home/medie/Rfile/chenchen/CCLM/4.zhushi')

Logfc = 0.5

Minpct = 0.35

DefaultAssay(CCLM) <- "RNA"

CCLM.markers <- FindAllMarkers(object = CCLM,logfc.threshold = Logfc,

min.pct = Minpct,only.pos = T)

CCLM.markers["pct.diff"]=CCLM.markers$pct.1-CCLM.markers$pct.2

CCLM.markers <- CCLM.markers[CCLM.markers$p_val_adj<0.05,]

length(unique(CCLM.markers$gene))

head(CCLM.markers)

diff.wilcox = FindAllMarkers(CCLM)

CCLM.markers = diff.wilcox %>% select(gene, everything()) %>% subset(p_val<0.05)

top10 = CCLM.markers %>% group_by(cluster) %>% top_n(n = 10, wt = avg_log2FC)

write.table(CCLM.markers,'scRNA_marker_gene1.txt',quote = F,row.names = F,sep='\t')

Top5 <- CCLM.markers %>%

group_by(cluster) %>%

slice_max(n =5, order_by = avg_log2FC)

write.table(Top5,'Top5.txt',quote = F,row.names = F,sep='\t')

Top5 <- unique(Top5$gene)

sc_marker_dotplot <- DotPlot(object = CCLM,

features = Top5,

cols=c("blue", "red"),

scale = T)+

RotatedAxis()+ ggtitle("Top 5 Marker Genes")+

theme(plot.title = element_text(hjust = 0.5))

sc_marker_dotplot

ggsave(filename = 'sc_marker_dotplot.pdf',

plot = sc_marker_dotplot,

height = 9,width = 25)

library(viridisLite)

sc_marker_heatmap<- DoHeatmap(object = CCLM,

features = Top5,

#group.colors = mycolor,

label = F) +

ggtitle("Top 5 Marker Genes") +

theme(plot.title = element_text(hjust = 0.5))

sc_marker_heatmap

ggsave(filename = 'sc_marker_heatmap5.pdf',

plot = sc_marker_heatmap,

width = 12,height = 24)

library(SingleR)

library(celldex)

library(BiocParallel)

library(Seurat)

library(viridis)

library(pheatmap)

testdata <- GetAssayData(CCLM, slot="data")

refdata <- HumanPrimaryCellAtlasData()

clusters <- CCLM@meta.data$seurat_clusters

cellpred <- SingleR(test = testdata, ref = refdata, labels = refdata$label.fine,

method = "cluster", clusters = clusters,

assay.type.test = "logcounts", assay.type.ref = "logcounts")

celltype = data.frame(ClusterID=rownames(cellpred), celltype=cellpred$labels, stringsAsFactors = F)

write.table(celltype,'celltype.txt',quote = F,sep = '\t',row.names = F)

CCLM@meta.data$celltype = "NA"

for(i in 1:nrow(celltype)){

CCLM@meta.data[which(CCLM@meta.data$seurat_clusters == celltype$ClusterID[i]),'celltype'] <- celltype$celltype[i]}

unique(CCLM$celltype)

library(paletteer)

paletteer_d("ggsci::nrc_npg")

allcolor<-paletteer_d("ggsci::nrc_npg")

allcolor

umap_celltype1<-DimPlot(CCLM,group.by = 'celltype',

cols = allcolor,

reduction="umap",

#reduction="tsne",

label = "F",

pt.size = 0.5,

label.size = 4)

umap_celltype1

ggsave('umap_celltype1111.pdf',umap_celltype1,he=7,wi=12)

colour=c("#7E0021FF","#F97B72FF")

umap_group1<-DimPlot(sce,group.by = 'Group1',

cols = colour,

#reduction="umap",

reduction="tsne",

label = "F",

pt.size = 0.2,

label.size = 4)

umap_group1

tsnem <- umap_celltype1|umap_group1

tsnem

table(CCLM$orig.ident)

table(CCLM$celltype)

table(CCLM$celltype, CCLM$orig.ident)

Cellratio <- prop.table(table(CCLM$celltype, CCLM$orig.ident), margin = 2)

Cellratio

write.table(Cellratio,'Cellratio.txt',quote = T,row.names = T,sep='\t')

Cellratio <- as.data.frame(Cellratio)

Cellratio

colourCount = length(unique(Cellratio$Var1))

library(ggplot2)

ratio <- ggplot(data=Cellratio,aes(x=Var2,y=Freq,fill=Var1)) +

geom_bar(stat="identity",position="stack") +

labs(x="Sample",y="Ratio") +

theme()

ratio

ratio1<-ratio+scale_fill_manual(values = c("#E64B35FF", "#4DBBD5FF", "#00A087FF", "#3C5488FF",

"#F39B7FFF", "#8491B4FF", "#91D1C2FF", "#DC0000FF",

"#7E6148FF", "#B09C85FF"))

ratio1

ggsave('ratio.pdf',ratio,he=7,wi=10)

save(CCLM,file = 'CCLM1.RData')

#load('sce1.RData')

setwd('/home/medie/Rfile/chenchen/CCLM/5.subcell')

rm()

Cells.sub_NK <- subset(CCLM@meta.data,

celltype == "NK_cell")

Cells.sub_NK <- subset(CCLM@meta.data,

celltype == "NK_cell"|“T_cell”)

CCLMnk_sce <- subset(CCLM, cells=row.names(Cells.sub_NK))

nk_sce = sce[,sce@meta.data$seurat_clusters %in% c(12)]`

save(CCLMnk_sce,file = 'CCLMnk_sce.RData')

load('CCLMnk_sce.RData')

CCLMnk_sce <- FindVariableFeatures(CCLMnk_sce, selection.method = "vst", nfeatures = 2000)

scale.genes <- rownames(CCLMnk_sce)

CCLMnk_sce <- ScaleData(CCLMnk_sce, features = scale.genes)

CCLMnk_sce <- RunPCA(CCLMnk_sce, features = VariableFeatures(CCLMnk_sce))

ElbowPlot(CCLMnk_sce, ndims=20, reduction="pca")

pc.num=1:15

CCLMnk_sce <- FindNeighbors(CCLMnk_sce, dims = pc.num)

CCLMnk_sce <- FindClusters(CCLMnk_sce, resolution = 0.1)

table(CCLMnk_sce@meta.data$seurat_clusters)

metadata <- CCLMnk_sce@meta.data

cell_cluster <- data.frame(cell_ID=rownames(metadata), cluster_ID=metadata$seurat_clusters)

write.csv(cell_cluster,'cell_cluster.csv',row.names = F)

CCLMnk_sce <- RunTSNE(CCLMnk_sce,

dims = pc.num,

reduction="pca")

embed_tsne <- Embeddings(CCLMnk_sce, 'tsne')

write.csv(embed_tsne,'embed_tsne.csv')

CCLMnk_sce <- RunUMAP(CCLMnk_sce, dims=pc.num, reduction="pca")

library(paletteer)

paletteer_d("ggthemes::gdoc")

allcolor<-paletteer_d("ggthemes::gdoc")

allcolor<-paletteer_d("ggsci::nrc_npg")

allcolor

plot1 = DimPlot(CCLMnk_sce, reduction = "tsne", cols = allcolor,

pt.size = 0.5,

label.size = 4)

plot1

diff.wilcox = FindAllMarkers(CCLMnk_sce)

all.markers = diff.wilcox %>% select(gene, everything()) %>% subset(p_val<0.05)

top50 = all.markers %>% group_by(cluster) %>% top_n(n = 50, wt = avg_log2FC)

Top10 <- all.markers %>%

group_by(cluster) %>%

slice_max(n =10, order_by = avg_log2FC)

write.table(top50,'Top50.txt',quote = F,row.names = F,sep='\t')

Top10 <- unique(Top10$gene)

NK_marker_dotplot <- DotPlot(object = CCLMnk_sce,

features = Top10,

cols=c("blue", "red"),

scale = T)+

RotatedAxis()+ ggtitle("Top 5 Marker Genes")+

theme(plot.title = element_text(hjust = 0.5))

NK_marker_dotplot <- DotPlot(object = CCLMnk_sce,

features = Top10,

group.by='seurat_clusters')

NK_marker_dotplot <- NK_marker_dotplot+scale_color_gradientn(values= seq(0,1,0.2), colours = c("#134B85","#2264A3", "#337CB7", "#4B94C4",

"#6BACD0","#8FC2DD", "#B1D4E7", "#CDE3EE",

"#E3EDF2","#F2EFEE", "#F9E8DD", "#FBD7C4",

"#F8BFA4","#F1A385", "#E48268", "#D56050",

"#C33D3D","#AC202F", "#8C0D25", "#67001F"))+theme_bw()

NK_marker_dotplot

ggsave(filename = 'NK_marker_dotplot.pdf',

plot = sc_marker_dotplot,

height = 9,width = 25)

p <- DotPlot(CCLMnk_sce, features=c('CD2', "GZMK", "NME8", "TEP1", "TRBC1","TTC38",

'CD69', "GRAP2", "SOCS1", "CCL4", "CCND2","APOL6",

'PTGER2', "NCR3", "OSM", "TNFSF14", "APOBEC3G","CDK6"), group.by='seurat_clusters')

p <- p + scale_color_gradientn(values= seq(0,1,0.2), colours = c("#134B85","#2264A3", "#337CB7", "#4B94C4",

"#6BACD0","#8FC2DD", "#B1D4E7", "#CDE3EE",

"#E3EDF2","#F2EFEE", "#F9E8DD", "#FBD7C4",

"#F8BFA4","#F1A385", "#E48268", "#D56050",

"#C33D3D","#AC202F", "#8C0D25", "#67001F"))+theme_bw()

p

library(viridisLite)

NK_marker_heatmap<- DoHeatmap(object = CCLMnk_sce,

features = Top10,

group.colors = allcolor,

label = F) +

ggtitle("Top 5 Marker Genes") +

theme(plot.title = element_text(hjust = 0.5))+

scale_fill_gradientn(colors = c("#377EB8", "white", "#E41A1C"))

NK_marker_heatmap

ggsave(filename = 'NK_marker_heatmap5.pdf',

plot = sc_marker_heatmap,

width = 12,height = 24)

write.table(top50,'top50.txt',quote = F,row.names = F,sep='\t')

write.table(all.markers,'nksce_marker2_gene.txt',quote = F,row.names = F,sep='\t')

write.csv(all.markers,'nksce_marker2_gene.csv',quote = F,row.names = F,sep='\t')

mycolor<-paletteer_d("nbapalettes::cavaliers_retro")

bfreaname.NK <- CCLMnk_sce

bfreaname.NK

new.cluster.id <- c("R", "R", "A", "R", "R", "A", "O", "O")

names(new.cluster.id) <- levels(CCLMnk_sce)

CCLMnk_sce <- RenameIdents(CCLMnk_sce, new.cluster.id)

plot4<-DimPlot(CCLMnk_sce,

group.by = 'Sample',

split.by = 'tissueunique'

#reduction="umap",

#cols = mycolor,

reduction="tsne",

label = "F",

pt.size = 2.0,

label.size = 4)

plot4

table(CCLMnk_sce$orig.ident)

table(CCLMnk_sce$seurat_clusters)

table(CCLMnk_sce$Group)

table(CCLMnk_sce$seurat_clusters, CCLMnk_sce$orig.ident)

Cellratio <- prop.table(table(CCLMnk_sce$seurat_clusters, CCLMnk_sce$orig.ident), margin = 2)

Cellratio

write.table(Cellratio,'nkratio.txt',quote = T,row.names = T,sep='\t')

Cellratio <- as.data.frame(Cellratio)

colourCount = length(unique(Cellratio$Var1))

setwd('/home/medie/Rfile/chenchen/6.nishixu')

library(monocle)

rm()

a<-paletteer_d("palettesForR::Browns")

a

select_genes <- c('CD8A')

p1 <- VlnPlot(CCLMnk_sce, features = select_genes, pt.size=0, ncol=2)

p1

ggsave("cell_identify/selectgenes_VlnPlot.png", p1, width=6 ,height=8)

mycolor <- c('WhiteSmoke', '#8B4513FF')

p2 <- FeaturePlot(CCLMnk_sce, features = select_genes, reduction = "umap", label=T, ncol=2,cols = mycolor)

p2

ggsave("cell_identify/selectgenes_FeaturePlot.png", p2, width=8 ,height=12)

p3=p1|p2

ggsave("cell_identify/selectgenes.png", p3, width=10 ,height=8)

set.seed(321)

sample(1:59529,30000,replace=F)->index

CCLMnk_sce2<-CCLMnk_sce[,index]

library(monocle)

library(clusterProfiler)

library(org.Hs.eg.db)

rm()

Logfc = 0.5

Minpct = 0.35

DefaultAssay(CCLMnk_sce2) <- "RNA"

CCLMnk_sce2.markers <- FindAllMarkers(object = CCLMnk_sce2,logfc.threshold = Logfc,

min.pct = Minpct,only.pos = T)

CCLMnk_sce2.markers["pct.diff"]=CCLMnk_sce2.markers$pct.1-CCLMnk_sce2.markers$pct.2

CCLMnk_sce2.markers <- CCLMnk_sce2.markers[CCLMnk_sce2.markers$p_val_adj<0.05,]

length(unique(CCLMnk_sce2.markers$gene))

head(CCLMnk_sce2.markers)

write.csv(CCLMnk_sce2.markers,'CCLMnk_sce2_marker_gene.csv',quote = F,row.names = F,sep='\t')

setwd('/home/medie/Rfile/chenchen/CCLM/6.nishixu')

load('CCLMnk_sce.RData')

library(clusterProfiler)

library(org.Hs.eg.db)

rm()

Logfc = 0.5

Minpct = 0.35

DefaultAssay(CCLMnk_sce) <- "RNA"

CCLMnk_sce.markers <- FindAllMarkers(object = CCLMnk_sce,logfc.threshold = Logfc,

min.pct = Minpct,only.pos = T)

CCLMnk_sce.markers["pct.diff"]=CCLMnk_sce.markers$pct.1-CCLMnk_sce.markers$pct.2

CCLMnk_sce.markers <- CCLMnk_sce.markers[CCLMnk_sce.markers$p_val_adj<0.05,]

length(unique(CCLMnk_sce.markers$gene))

head(CCLMnk_sce.markers)

write.csv(CCLMnk_sce.markers,'CCLMnk_sce_marker2_gene.csv',quote = F,row.names = F,sep='\t')

dir.create("pseudotime")

data <- as(as.matrix(CCLMnk_sce2@assays$RNA@counts), 'sparseMatrix')

pd <- new('AnnotatedDataFrame', data = CCLMnk_sce2@meta.data)

fData <- data.frame(gene_short_name = row.names(data), row.names = row.names(data))

fd <- new('AnnotatedDataFrame', data = fData)

mycds <- newCellDataSet(data,

phenoData = pd,

featureData = fd,

expressionFamily = negbinomial.size())

mycds <- estimateSizeFactors(mycds)

mycds <- estimateDispersions(mycds, cores=4, relative_expr = TRUE)

var.genes <- VariableFeatures(CCLMnk_sce)

mycds <- setOrderingFilter(mycds, var.genes)

p2 <- plot_ordering_genes(mycds)

p2

disp_table <- dispersionTable(mycds)

disp.genes <- subset(disp_table, mean_expression >= 0.1 & dispersion_empirical >= 1 * dispersion_fit)$gene_id

mycds <- setOrderingFilter(mycds, disp.genes)

p3 <- plot_ordering_genes(mycds)

p3

diff.genes <- read.csv('CCLMnk_sce2_marker_gene.csv')

diff.genes

diff.genes <- subset(diff.genes,p_val_adj<0.001)$gene

mycds <- setOrderingFilter(mycds, diff.genes)

p1 <- plot_ordering_genes(mycds)

p1

ggsave("p1.png", plot = plot1, width = 8, height = 7)

p1|p2|p3

mycds <- reduceDimension(mycds, max_components = 2, method = 'louvain')

mycds <- orderCells(mycds)

State <- plot_cell_trajectory(mycds, color_by = "State")

State

ggsave("pseudotime/State.pdf", plot = State, width = 6, height = 5)

Cluster <- plot_cell_trajectory(mycds, color_by = "Group")

Cluster

ggsave("pseudotime/Cluster.pdf", plot = Cluster, width = 6, height = 5)

Logfc = 0.5

Minpct = 0.35

DefaultAssay(CCLMnk_sce2) <- "RNA"

CCLMnk_sce2.markers <- FindAllMarkers(object = CCLMnk_sce2,logfc.threshold = Logfc,

min.pct = Minpct,only.pos = T)

nk_sce.markers["pct.diff"]=nk_sce.markers$pct.1-sce.markers$pct.2

nk_sce.markers <- nk_sce.markers[nk_sce.markers$p_val_adj<0.05,]

length(unique(nk_sce.markers$gene))

head(nk_sce.markers)

write.csv(nk_sce.markers,'scRNA_marker2_gene.csv',quote = F,row.names = F,sep='\t')

dir.create("pseudotime")

data <- as(as.matrix(nk_sce@assays$RNA@counts), 'sparseMatrix')

pd <- new('AnnotatedDataFrame', data = nk_sce@meta.data)

fData <- data.frame(gene_short_name = row.names(data), row.names = row.names(data))

fd <- new('AnnotatedDataFrame', data = fData)

mycds <- newCellDataSet(data,

phenoData = pd,

featureData = fd,

expressionFamily = negbinomial.size())

mycds <- estimateSizeFactors(mycds)

mycds <- estimateDispersions(mycds, cores=4, relative_expr = TRUE)

var.genes <- VariableFeatures(nk_sce)

mycds <- setOrderingFilter(mycds, var.genes)

p2 <- plot_ordering_genes(mycds)

p2

disp_table <- dispersionTable(mycds)

disp.genes <- subset(disp_table, mean_expression >= 0.1 & dispersion_empirical >= 1 * dispersion_fit)$gene_id

mycds <- setOrderingFilter(mycds, disp.genes)

p3 <- plot_ordering_genes(mycds)

p3

diff.genes <- read.csv('nksce_marker2_gene.csv')

diff.genes

diff.genes <- subset(diff.genes,p_val_adj<0.01)$gene

mycds <- setOrderingFilter(mycds, diff.genes)

p1 <- plot_ordering_genes(mycds)

p1

ggsave("p1.png", plot = plot1, width = 8, height = 7)

p1|p2|p3

mycds <- reduceDimension(mycds, max_components = 2, method = 'DDRTree')

mycds <- orderCells(mycds)

State <- plot_cell_trajectory(mycds, color_by = "State")

State

ggsave("pseudotime/State.pdf", plot = plot1, width = 6, height = 5)

Cluster <- plot_cell_trajectory(mycds, color_by = "Group")

Cluster

ggsave("pseudotime/Cluster.pdf", plot = plot2, width = 6, height = 5)

Pseudotime <- plot_cell_trajectory(mycds, color_by = "Pseudotime")

Pseudotime

ggsave("pseudotime/Pseudotime.pdf", plot = plot3, width = 6, height = 5)

plotc <- plot1|plot2|plot3

plotc

ggsave("pseudotime/Combination.pdf", plot = plotc, width = 10, height = 3.5)

ggsave("pseudotime/Combination.png", plot = plotc, width = 10, height = 3.5)

write.csv(pData(mycds), "pseudotime/pseudotime.csv")

p1 <- plot_cell_trajectory(mycds, color_by = "State") + facet_wrap(~State, nrow = 1)

p2 <- plot_cell_trajectory(mycds, color_by = "seurat_clusters") + facet_wrap(~seurat_clusters, nrow = 1)

plotc <- p1/p2

plotc

ggsave("pseudotime/trajectory_facet.png", plot = plotc, width = 6, height = 5)

diff.genes <- read.csv('scRNA_marker2_gene.csv')

sig_diff.genes <- subset(diff.genes,p_val_adj<0.0001&abs(avg_log2FC)>0.75)$gene

sig_diff.genes <- unique(as.character(sig_diff.genes))

diff_test <- differentialGeneTest(mycds[sig_diff.genes,], cores = 1,

fullModelFormulaStr = "~sm.ns(Pseudotime)")

sig_gene_names <- row.names(subset(diff_test, qval < 0.01))

p1 = plot_pseudotime_heatmap(mycds[sig_gene_names,], num_clusters=3,

show_rownames=T, return_heatmap=T)

ggsave("pseudotime/pseudotime_heatmap1.png", plot = p1, width = 5, height = 8)

disp_table <- dispersionTable(mycds)

disp.genes <- subset(disp_table, mean_expression >= 0.5&dispersion_empirical >= 1*dispersion_fit)

disp.genes <- as.character(disp.genes$gene_id)

diff_test <- differentialGeneTest(mycds[disp.genes,], cores = 4,

fullModelFormulaStr = "~sm.ns(Pseudotime)")

sig_gene_names <- row.names(subset(diff_test, qval < 1e-04))

p2 = plot_pseudotime_heatmap(mycds[sig_gene_names,], num_clusters=5,

show_rownames=T, return_heatmap=T)

ggsave("pseudotime/pseudotime_heatmap2.png", plot = p2, width = 5, height = 10)

disp_table <- dispersionTable(mycds)

disp.genes <- subset(disp_table, mean_expression >= 0.5&dispersion_empirical >= 1*dispersion_fit)

disp.genes <- as.character(disp.genes$gene_id)

mycds_sub <- mycds[disp.genes,]

plot_cell_trajectory(mycds_sub, color_by = "State")

beam_res <- BEAM(mycds_sub, branch_point = 1, cores = 8)

beam_res <- beam_res[order(beam_res$qval),]

beam_res <- beam_res[,c("gene_short_name", "pval", "qval")]

mycds_sub_beam <- mycds_sub[row.names(subset(beam_res, qval < 1e-4)),]

plot_genes_branched_heatmap(mycds_sub_beam, branch_point = 1, num_clusters = 3, show_rownames = T)

rm()

library(Seurat)

library(ggplot2)

library(patchwork)

library(dplyr)

library(cowplot)

setwd('/home/medie/Rfile/chenchen/space/ST/ST-colon1')

name='colon1'

expr <- "/home/medie/Rfile/chenchen/space/ST/ST-colon1/filtered_feature_bc_matrix.h5"

expr.mydata <- Seurat::Read10X_h5(filename = expr )

mydataC1 <- Seurat::CreateSeuratObject(counts = expr.mydata, project = 'colon1', assay = 'Spatial')

mydataC1$slice <- 1

mydataC1$region <- 'colon1'

imgpath <- "/home/medie/Rfile/chenchen/space/ST/ST-colon1"

img <- Seurat::Read10X_Image(image.dir = imgpath)

Seurat::DefaultAssay(object = img) <- 'Spatial'

img <- img[colnames(x = mydata)]

mydataC1[['image']] <- img

mydataC1

C1plot1 <- VlnPlot(mydataC1, features = "nCount_Spatial", pt.size = 0.5) + NoLegend()

C1plot1

C1plot2 <- SpatialFeaturePlot(mydataC1, features = "nCount_Spatial") + theme(legend.position = "right")

C1plot2

C1plot3 <- VlnPlot(mydataC1, features = "nFeature_Spatial", pt.size = 0.5) + NoLegend()

C1plot3

C1plot4 <- SpatialFeaturePlot(mydataC1, features = "nFeature_Spatial") + theme(legend.position = "right")

C1plot4

mydataC1[["percent.mt"]] <- PercentageFeatureSet(mydataC1, pattern = "^MT[-]")

C1plot5 <- VlnPlot(mydataC1, features = "percent.mt", pt.size = 0.5) + NoLegend()

C1plot5

C1plot6 <- SpatialFeaturePlot(mydataC1, features = "percent.mt") + theme(legend.position = "right")

C1plot6

mydataC1 <- subset(mydataC1, subset = nFeature_Spatial > 200 & nFeature_Spatial <7500 & nCount_Spatial > 1000 & nCount_Spatial < 60000 & percent.mt < 25)

mydataC1

mydataC1 <- SCTransform(mydataC1, assay = "Spatial", verbose = FALSE)

top10 <- head(VariableFeatures(mydataC1),10)

top10

mydataC1 <- RunPCA(mydataC1, assay = "SCT", verbose = FALSE)

mydataC1 <- FindNeighbors(mydataC1, reduction = "pca", dims = 1:30)

mydataC1 <- FindClusters(mydataC1, verbose = FALSE)

mydataC1 <- RunUMAP(mydataC1, reduction = "pca", dims = 1:30)

mydataC1 <- RunTSNE(mydataC1, reduction = "pca",dims = 1:30)

C1p9 <- DimPlot(mydataC1, reduction = "tsne", label = TRUE)

C1p9

C1p10 <- SpatialDimPlot(mydataC1, label = TRUE, label.size = 3)

C1p10

data.markersC1 <- FindAllMarkers(mydataC1, only.pos = FALSE, min.pct = 0.25, logfc.threshold = 0.25,test.use = "wilcox")

data.markersC1

write.table(data.markersC1,'data.markersC1.txt',quote = F,row.names = F,sep='\t')

topgeneC1<-data.markersC1 %>% group_by(cluster) %>% top_n(n = 5, wt = avg_log2FC)

topgeneC1

DoHeatmap(mydataC1, features = topgeneC1$gene,size = 2) + NoLegend()

library(SingleR)

library(celldex)

library(BiocParallel)

library(Seurat)

library(viridis)

library(pheatmap)

ref.se <- HumanPrimaryCellAtlasData()

expdata =mydata2[["Spatial"]]@data

clusters <- mydata2@meta.data$seurat_clusters

clusters

anno.cluster.main<- SingleR(test = expdata, ref = ref.se, labels = ref.se$label.fine, method= "cluster", clusters = clusters)

DimPlot(mydata2,reduction = "tsne" ,group.by="celltype")

SpatialDimPlot(mydata2, label = F, label.size = 3,group.by="celltype")

allcolour=c("#FF34B3", "#BC8F8F", "#20B2AA", "#00F5FF", "#FFA500", "#ADFF2F", "#FF6A6A", "#7FFFD4",

"#AB82FF", "#90EE90", "#00CD00", "#008B8B", "#6495ED", "#FFC1C1", "#CD5C5C", "#8B008B",

"#FF3030", "#7CFC00", "#000000", "#708090", "#DC143C","#0000FF","#20B2AA","#FFA500","#9370DB","#98FB98","#F08080","#1E90FF","#7CFC00","#FFFF00",

"#808000","#FF00FF","#CCCCFF","#000000","#7B68EE","#9400D3","#A0522D","#800080","#D2B48C","#D2691E",

"#87CEEB","#40E0D0","#5F9EA0","#FF1493","#0000CD","#008B8B","#FFE4B5","#8A2BE2","#228B22","#E9967A",

"#4682B4","#32CD32","#F0E68C","#FFFFE0","#EE82EE","#FF6347","#6A5ACD","#9932CC","#8B008B","#8B4513",

"#DEB887")

celltype = data.frame(ClusterID=rownames(anno.cluster.main), celltype=anno.cluster.main$labels, stringsAsFactors = F)

celltype

mydata2[['celltype']]<-

celltype$celltype[match(Idents(mydata2), celltype$ClusterID)]

DimPlot(mydata2,reduction = "tsne" ,group.by="SingleR.labels",cols = allcolour)

head(mydata2@meta.data)

SpatialDimPlot(mydata2, label = F, label.size = 3,group.by="SingleR.labels")

anno.cell.main=SingleR(test=expdata , ref = ref.se, labels = ref.se$label.main)

mydata2[["SingleR.labels"]]<- as.character(anno.cell.main$labels)

DimPlot(mydata2,reduction = "tsne" ,group.by="SingleR.labels")

NKA <- list(c("APOBEC3G","APOL6","CCND2","CCL4","CD69","CDK6","CSF2",

"DPP4","FASLG","GPR171","GPR18","GRAP2","IFNG","KIR2DL4",

"KIR2DS4","LTA","LTB","NCR3","OSM","PTGER2","SOCS1","TNFSF14"))

NKA <- list(c("TPSB2","TPSAB1","CPA3","LTC4S","CD69","CLU","HPGDS",

"CTSG","CD63","ANXA1"))

mydata3 <- AddModuleScore(mydata2,features = NKA, ctrl = 100, name = "NKA")

head(mydata3@meta.data)

colnames(mydata3@meta.data)

colnames(mydata3@meta.data)[7] <- "NKA"

VlnPlot(mydata3,features = "NKA")

plot1<-SpatialFeaturePlot(mydata3, features = "NKA")

plot1

NKR <- list(c("AZU1","BPI","CAMP","CD160","CDHR1","CEACAM8",

"DEFA4","ELANE","GFI1","GZMK","KLRC4","MGAM","MS4A3",

"NME8","PLEKHF1","TEP1","TTC38","ZNF135"))

NKR <- list(c("AZU1","CDHR1","DEFA4","ELANE","PLEKHF1","TEP1","TTC38","ZNF135"))

mydata4 <- AddModuleScore(mydata2,features =NKR, ctrl = 100, name = "NKR")

head(mydata4@meta.data)

colnames(mydata4@meta.data)

colnames(mydata4@meta.data)[13] <- "NKR"

VlnPlot(mydata4,features = "NKR")

plot2<-SpatialFeaturePlot(mydata4, features = "NKR")

wrap_plots(plot1, plot2)
